# Clinical Evidence and Practice-Based Guidelines on the Utility of Basal Insulin Combined Oral Therapy (Metformin and Glimepiride) in the Current Era

**DOI:** 10.2174/1573399819666230109104300

**Published:** 2023-08-02

**Authors:** Abhishek Shrivastava, Jothydev Kesavadev, Viswanathan Mohan, Banshi Saboo, Dina Shrestha, Anuj Maheshwari, Brij Mohan Makkar, Kirtikumar D. Modi, Ashok Kumar Das

**Affiliations:** 1 R&R Hormone Clinic, Jabalpur, Madhya Pradesh, India;; 2 Jothydev's Diabetes Research Centre, Konkalam Road, Mudavanmugal, Trivandrum, Kerala, India;; 3 Dr. Mohan’s Diabetes Specialities Centre and Madras Diabetes Research Foundation, Chennai, India;; 4 Diabetes Care and Hormone Clinic, Ahmedabad, Gujarat, India;; 5 Norvic International Hospital and Medical College, and Hospital for Advanced Medicine and Surgery, Maharajganj, Kathmandu, Nepal;; 6 Department of Medicine, American College of Physicians, BBD University, Lucknow, India;; 7 Dr. Makkar’s Diabetes & Obesity Centre, Paschim Vihar, New Delhi, India;; 8 Care Hospital, Hyderabad, Telangana, India;; 9 Pondicherry Institute of Medical Sciences, Puducherry, India

**Keywords:** Degludec, glargine, glimepiride, metformin, oral, anti-diabetic drugs

## Abstract

**Background and Aim:**

Basal insulin combined oral therapy consisting of insulin and oral anti-diabetic drugs (OADs) is recommended for type 2 diabetes uncontrolled on OADs. There is a lack of clear evidence and recommendations on the combined use of basal insulin analogues to more than one OADs (glimepiride plus metformin) in effective control of glycemic parameters and its safety in terms of reduced hypoglycemic events, weight gain and cardiovascular risk. In this context, a group of clinical experts discussed the utility of basal insulin combined oral therapy with metformin and glimepiride in the current era.

**Methods:**

The clinical experts discussed and provided their inputs virtually. The expert panel included clinical experts comprising endocrinologists and diabetologists from India and Nepal.

**Results:**

The panel thoroughly reviewed existing literature on the subject and proposed clinical evidence and practice-based guidelines.

**Conclusion:**

These current clinical practice guidelines highlight the efficacy and safety of basal insulin combination therapy with various available basal insulins including neutral protamine hagedorn, detemir, glargine and degludec in addition to metformin and glimepiride therapy.

## 1 INTRODUCTION

Type 2 diabetes mellitus (T2DM) may initially be controlled through dietary modifications and exercise; further oral antidiabetic agents (OADs) help to maintain glycemic control and they are one of the mainstays of management of T2DM [1]. In spite of multiple options of OADs (including sulfonylureas (SUs), biguanides, meglitinides, dipeptidyl peptidase-4 inhibitors [DPP4i]), sodium glucose cotransporter 2 inhibitor [SGLT2i]) available in the current market, the therapeutic strategies are still evolving to establish an ideal oral therapy in the management of T2DM. Metformin and glimepiride are the most commonly used first- and second-line therapies in T2DM in India [2]. However, patients may eventually need insulin therapy as a result of advancing disease to achieve optimal glycemic control [1]. In recent times, the use of basal insulin combined oral therapy in patients with uncontrolled glycemic parameters on OADs has remarkably increased. Evidence suggests that combination therapy with insulins and OADs initiation before depletion of pancreatic β cells might enhance its effectiveness in achieving glycemic control [3]. Basal insulin combined oral therapy is also accompanied by several benefits such as reduced insulin dose and injection frequency, and decreased hypoglycemia events and blood glucose monitoring as compared with basal-bolus insulin therapy [4, 5]. Furthermore, this combination therapy provides greater flexibility in meal frequency [6]. Basal insulin analogues effectively lower the glycated hemoglobin (HbA1c) levels and therefore are added to the regimen of patients with inadequately controlled glycemia using OADs or non-insulin therapies [7]. Factors that may predict the initiation of basal insulin combined oral therapy for patients with T2DM, who were initially prescribed one or more OADs, have been identified. These factors include age, gender, long duration of T2DM, increased body mass index, poor glycemic control, micro- or macro-vascular comorbidity, concomitant non-antidiabetic medications, and prescribed OADs [8]. Data from clinical studies support the use of bedtime insulin combined with more than one OADs for patients with inadequately controlled glycemia with other therapeutic interventions [9]. The bedtime insulin daytime SU therapy uses insulin dosing based on the pathophysiology of fasting hyperglycemia in patients with T2DM. This combination therapy is based on the rationale that using evening insulin decreases fasting blood glucose (FPG) level to normal, thus improving glycemic control with daytime SUs or any other OAD throughout the day [10]. Therefore, use of this combination therapy has the potential to achieve glycemic control in patients with T2DM with poor glycemic control. To the best of our knowledge, currently there are no recommendations particularly focusing on clinical aspects of basal insulin combined oral therapy (metformin+ glimepiride) in T2DM management. In this regard, a group of clinical experts discussed and proposed recommendations on the efficacy and safety of basal insulin combined oral therapy comprising metformin and glimepiride in patients with T2DM.

## 2 COMBINATION THERAPY OF METFORMIN AND GLIMEPIRIDE

**Fig. (1):**
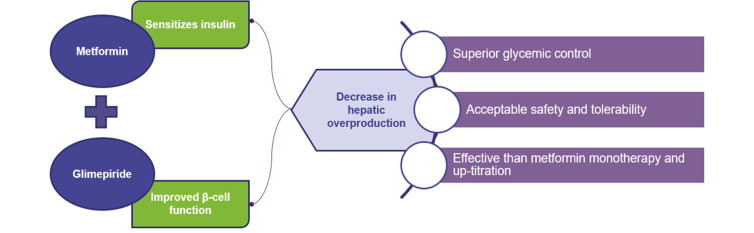
Synergistic mechanism of action and clinical benefits of metformin and glimepiride combination [11].

Metformin improves sensitivity to insulin, while glimepiride augments the β-cell sensitivity to glucose and enables endogenous insulin secretion [11]. Glimepiride and metformin together present a complementary mechanism of action resulting in a significant reduction of glycemic parameters (FPG, postprandial plasma glucose [PPG], and HbA1c levels) [12] (Fig. **1**). Glimepiride has several advantages, including optimal insulin secretion, extra pancreatic effects, enhanced beta-cell function, weight-neutral effects, absence of cardiovascular risk, and reduced hypoglycemic events compared to older generation SUs [13]. In comparison to other antidiabetic agents such as pioglitazone [14] and DPP4i, glimepiride showed favorable glycemic control [15]. Given the advantages of glimepiride, it is a rational choice to be used as an add-on therapy with metformin. There are multiple strengths of this combination therapy commonly prescribed by clinicians in India [16, 17].

## 3 CURRENTLY USED BASAL INSULIN ANALOGUES

Basal insulin is a convenient and effective option in the majority of patients, provided the risk of post-meal hyperglycemia is not elevated significantly. Table **1** summarizes the key properties of different basal insulin preparations. Clinical trials have explored and compared the safety and efficacy profile of basal insulin neutral protamine hagedorn (NPH), glargine-100 U/mL/ 300 U/mL (Gla-100/Gla-300), degludec and detemir. A review summarized the data on the safety and efficacy of newer basal insulin analogues compared to the conventional basal insulin and premixed insulin. The newer analogues have better pharmacokinetic and pharmacodynamic profiles, hence a higher compliance rate and improved quality of life of patients with T2DM [18]. The safety profile of basal insulin analogues is remarkable in terms of reducing hypoglycemia risk, particularly nocturnal hypoglycemia [19]. In comparison to premixed insulin, basal insulin has lower rates of hypoglycemia and higher dosing flexibility [20]. Basal insulins have superior glycemic benefits and acceptable tolerability compared to Basal insulins have superior glycemic benefits and acceptable tolerability compared to premixed insulin. Moreover, the GINGER study showed superior glycemic control without an increase in hypoglycemia using insulin Gla-100 than premixed insulin in patients with long-standing T2DM [21].

Clinical studies have shown that once-daily administration of Gla-100 with OADs resulted in a significant reduction of HbA1c levels with minimal risk of hypoglycemia [22]. Additionally, Gla-100 is observed to have a neutral effect on cardiovascular outcomes with near-normal glycemic control and delayed dysglycemia progression as confirmed by the ORIGIN trial [23]. Essentially, Gla-100 is commonly used for combination therapy, with approximately 29% of patients using it in combination with one OAD, 30% with two OADs, and 21% with three OADs. In addition, combination therapy of Gla-100 with OADs was well-tolerated, and showed improved treatment satisfaction and self-reported health [24]. A study conducted by Seufert J *et al.* [25] demonstrated that switching the basal insulin to Gla-300 in basal insulin supported oral regimen improved metabolic control and treatment satisfaction with a decreased risk of symptomatic and nocturnal hypoglycemia and without weight gain. It is evident that insulin detemir implemented as an add-on therapy to OADs improved glycemic control without increased risk of hypoglycemia, adverse events or body weight compared with baseline [26].

A patient level meta-analysis of EDITION 1,2 and 3 studies stated that although Gla-100 and Gla-300 showed a comparable glycemic profile, with less hypoglycemia at any time of day (24 h) and a more pronounced reduction in nocturnal hypoglycemia [27]. Insulin Gla-100 and detemir have a significantly lower rate of hypoglycemia compared to NPH while the second-generation long-acting basal insulin preparations, degludec and the Gla-300, are superior in terms of pharmacokinetic and pharmacodynamic properties as compared to the currently available first-generation basal insulin preparations [28]. Rosenstock *et al.* [29] demonstrated in the BRIGHT study that Gla-300 and IDeg-100 have similar glycemic control, hypoglycemia incidence and rates were comparable with both insulins during the full study period but lower in favor of Gla-300 during the titration period.

## 4 COMBINATION THERAPY OF METFORMIN AND GLIMEPIRIDE PLUS BASAL INSULINS

In patients with T2DM, poor glycemic parameters, vascular comorbidities, concomitant medications, and a higher number of OADs enhance the probability of a switch to basal combined oral therapy (BOT). Sulfonylurea (71.5%), DPP4i (60.7%), and biguanide (48.6%) are frequently administered OADs in BOT [30]. A cohort study that followed up a series of 194,967 OAD users showed that 12.8% of patients switched from OAD therapy to basal combined oral therapy during the observational period indicating the key role of BOT in controlling glycemic parameters [8]. A randomized controlled trial showed that addition of glimepiride to on-going treatment of metformin and insulin in patients with T2DM for more than 10 years, lowered HbA1c levels and/or reduced the need for exogenous insulin [31]. However, a real-world study reported a contradictory observation that the likelihood of attaining glycemic control with basal insulin after OADs diminished over time and remained low from 12 months onwards. Further, this study also suggested that maintaining basal insulin therapy offered benefits in attaining glycemic target [32]. Therefore, BOT seems to offer effective glycemic control in uncontrolled T2DM.

Comparison of the efficacy and safety of combined therapy consisting of either Gla-100 plus glimepiride and metformin 1/500 mg twice daily or Gla-100 plus glimepiride 4 mg once daily indicated that both forms of therapy were relatively safe; however, the former BOT more effectively lowered HbA1c and blood glucose levels [33]. In another clinical study conducted on patients with uncontrolled T2DM who were taking SU and metformin and received either premixed insulin, bolus insulin, or basal insulin analogue, the median HbA1c levels were similar among the groups [34]. Initiating Gla-100 in patients with T2DM, previously uncontrolled on OADs (metformin or SU, or both) demonstrated early and sustained glycemic benefits with a low-risk of hypoglycemia [35]. Use of Gla-100 with glimepiride plus metformin showed a significantly higher reduction in HbA1c levels than with Gla-100 plus metformin (0.49% [CI, 0.16% to 0.82%]; P=0.005) and Gla-100 plus glimepiride (0.59% [CI, 0.13% to 1.05%]; *P =* 0.012) [36]. The patients with T2DM who failed to achieve target HbA1c level with previous premixed insulin with or without OAD (glimepiride plus metformin) showed that switching to a combination therapy of Gla-100 and glimepiride improved glycemic control along with high patient tolerability [6]. A pilot study was conducted to determine whether Gla-100 plus OADs is effective in patients with T2DM previously on premixed insulin therapy and inadequately controlled. A significant decrease in HbA1c level was observed from baseline in the Gla-100 plus glimepiride group and Gla-100 plus glimepiride and metformin group, but not in the premixed insulin group. The results showed no between-treatment differences at the endpoint in HbA1c, FPG, mean daily blood glucose or hypoglycemia [37]. However, patients with T2DM inadequately controlled with premixed insulin showed significant improvements in glycemic parameters post-switching to Gla-100 plus OADs (majority with metformin and glimepiride) [38]. Thus, BOT comprising metformin and glimepiride might be a suitable treatment approach in patients with uncontrolled T2DM. Table **2** summarizes the efficacy and safety outcomes from recent clinical studies of insulin and metformin and glimepiride combination in patients with T2DM.

The dual combination of glimepiride and insulin upsurges insulin levels leading to insulin-sparing effect and lowering the daily dose of insulin. Glimepiride increases the serum levels of high molecular weight adiponectin resulting in improved glycemic control [39]. Glimepiride is the only Food and Drug Administration (FDA) approved SU indicated to be used in combination with insulin [13]. A study conducted in patients with poorly controlled T2DM demonstrated that adding glimepiride to current insulin treatment led to a significant reduction in HbA1c, FPG and PPG levels. Further, glimepiride added to insulin group showed a significant decrease in insulin doses, incidence of hypoglycemia and no weight gain [39]. In another study, treating with insulin only and in combination with glimepiride showed no difference in hypoglycemic events in patients with T2DM. However, glimepiride plus insulin improved glycemic control in patients with poorly controlled T2DM [40]. Although metformin is the gold standard for treating T2DM as endorsed by several guidelines, yet a great proportion of patients cannot tolerate metformin due to associated gastrointestinal intolerance [41]. Thus, glimepiride plus BOT may be considered as the potential option of combination therapy in metformin intolerant patients.

## 5 SAFETY: HYPOGLYCEMIA AND WEIGHT GAIN

Initiation and aggressive titration of basal insulin combined with oral therapy in patients with suboptimal control of T2DM help attain guideline recommended HbA1c levels in majority of patients.

Metformin, in combination with insulin, limits the risk of weight gain [42]. Clinical evidence showed better outcomes with glimepiride in those with underlying coronary artery disease. Increased overall mortality risk was observed with glyburide or glipizide than with glimepiride in patients with cardiovascular disease [43]. Glimepiride in combination with morning or bedtime Gla-100 showed lowered risk for nocturnal hypoglycemia in patients with T2DM who were previously treated with OADs [44]. Glimepiride and metformin showed favorable safety and tolerability in terms of reducing hypoglycemia, weight gain, and cardiovascular profile as compared to older generation SUs [11]. Weight gain and the risk of hypoglycemia did not significantly differ among the groups treated with Gla-100 plus glimepiride and metformin combination therapy and Gla-100 plus metformin alone [36]. Furthermore, once-daily basal Gla-100 along with glimepiride and metformin therapy, was observed to be safer and more effective than twice-daily injections of 70/30 premixed insulin without OADs in patients with inadequately controlled T2DM [45]. Evidence suggests that hypoglycemia is more common in individuals treated with premixed and bolus, and weight gain is more common in those treated with bolus; while in patients treated with BOT, fewer adverse effects were observed compared with those treated with premixed or bolus insulin [34].

## 6 IMPLEMENTING BASAL INSULIN COMBINED ORAL THERAPY IN SPECIAL POPULATIONS

Guidelines and clinical studies recommend metformin and glimepiride administration in patients with T2DM who are elderly and during fasting due to the lower risk of hypoglycemia associated with their use [46]. However, limited data exist for initiating BOT in these populations.

Second-generation basal insulin (BI) analogues (Gla-300, degludec) have comparable glycemic efficacy with less hypoglycemia compared to first generation BI analogues [47]. The SENIOR study investigated the efficacy and safety of Gla-300 *vs*. Gla-100, this study demonstrated that Gla-300 has good efficacy and safety in older people with type 2 diabetes, particularly in people with ≥75 years age group [48]. A real-world study from Switzerland estimated the effectiveness of Gla-300 in elderly patients with uncontrolled T2DM. It is reported that upon switching the first-generation basal insulin to second-generation basal insulin, Gla-300, the overall glucose control was significantly improved, and glycemic targets were achieved with reduced hypoglycemic episodes in these patients [49]. A meta-analysis of seven BEGIN trials showed a lower risk of overall confirmed hypoglycemia in elderly patients with T2DM receiving insulin degludec compared with Gla-100 [50].

Patients with T2DM are highly prone to increased rates of hypoglycemia during fasting in Ramadan. Guidelines developed for managing T2DM in patients during Ramadan recommend basal insulin analogues due to low risk of hypoglycemia than with regular human insulin [51, 52]. The ORION study showed safe and effective use of Gla-300 with low hypoglycemia and improved glycemic parameters in patients who fasted during Ramadan [53].

Patients with chronic kidney failure having T2DM are often difficult to treat due to higher rates of hypoglycemic events. Also, evidence confirming ideal insulin therapy in such patients is scarce owing to the lack of pharmacokinetic analysis for various insulin preparations. Treating with Gla-100 based basal insulin therapy in T2DM with renal failure is safe and efficacious in decreasing glycemic parameters without significant weight gain or hypoglycemia [54]. A subgroup analysis of BRIGHT trial comparing Gla-300 and degludec stated that greater HbA1c reductions with Gla-300 without increase in hypoglycemia risk in patients with eGFR <60 mL/min/1.73 m^2^ [55]. Nevertheless, further studies are essential in special patient populations to compare and confirm the possible beneficial effect of different basal insulins.

## 7 PRECAUTIONS TO BE TAKEN WHILE USING BASAL INSULIN SUPPORTED ORAL THERAPY

It is recommended to start basal insulin combination therapy at a low dose whenever SU is employed. The dosage can be increased at intervals of 2-4 weeks until the glycemic target is reached [56]. Regular monitoring is recommended, especially when combining basal insulin with SUs for timely alterations if required. If glycemic control is not achieved with once-daily dosing, accurate titration of the optimal basal insulin dose should be done by a clinician before intensification of the insulin regimen (Table **3**).

## CONCLUSION

The addition of newer basal insulin analogues to more than one OADs (glimepiride plus metformin) seems to be effective in controlling glycemic parameters and safe in terms of hypoglycemic events, weight gain, and cardiovascular risk. This combination regimen will contribute to lowering insulin dosages and injection frequency. Precautions to be taken when using a basal combination therapy with glimepiride and metformin to avoid hypoglycemia. Initiatives should be taken to build a concrete guideline for BOT implementation to guide its application in routine clinical practice.

## AUTHORS’ CONTRIBUTIONS

All authors critically reviewed the manuscript and provided approval on the final draft being submitted and took complete responsibility of data accuracy. All authors met the International Committee of Medical Journal Editors (ICMJE) authorship criteria and all those who met the ICMJE authorship criteria are listed as authors. We thank Dr. Rajshri Mallabadi from BioQuest Solutions Pvt. Ltd., Bangalore, for providing medical writing assistance and editorial support in the preparation of this manuscript. We also thank Dr. Amarnath S for editorial and scientific support.

## Figures and Tables

**Table 1:** Key properties of different basal insulin preparations.

**Basal Insulin Class**	**Insulin Preparation**	**Duration of Action (Hours)**	**Properties**
Intermediate-acting insulin	Neutral protamine hagedorn insulin	8-12	Gradual onset of action, the greatest risk for hypoglycemia Requires twice-daily injection Preferred during pregnancy
Long-acting first-generation insulin	Detemir	16-24	It can be dosed once or twice a day. It has low interpatient variability and a reduced risk of hypoglycemia as compared to NPH
	Glargine-100	>24	It is widely used Usually injected once a day that begins working a few hours after injection It can be administered anytime each day
Long-acting second-generation insulin	Glargine-300	>24	It has a lesser intra-/inter-variability It has lower risk of nocturnal hypoglycemia, a slightly smaller weight gain and better flexibility in dosing time
	Degludec	>24	It is an ultralong-acting with a long half-life, injecting once daily and a consistent glucose-lowering effect Lower risk of hypoglycemia

**Note:** [60-63]

**Table 2:** Summary of efficacy and safety outcomes from recent clinical studies of insulin and metformin and glimepiride combination in patients with T2DM.

**Author/** **References**	**Intervention**	**Mean Change in HbA1c Levels/ HbA1c at Baseline *vs.* Endpoint (%)**	**Fasting Blood Glucose Level** **(mg/dL)**	**Post Prandial Blood Glucose Level** **(mg/dL)**	**Hypoglycemia Events (%)**	**Body Weight/BMI Change (Kg)**
Cerghizan *et al*. [57] (2020)	Newly initiated with basal insulin therapy *vs.* treated for less than 12 months with basal insulin + OADs	-1.4 *vs.* -0.75	NR	NR	9.7 *vs.* 12.8	NR
Yu *et al.* [33] (2019)	Insulin glargine + a fixed-dose glimepiride/metformin *vs.* insulin glargine + glimepiride	-0.99 *vs.* -0.20 (P<0.0001)	-53.3 *vs.* -37.6 (P=0.1807)	-70.6 *vs.* -36.9 (P=0.0349)	79 events in 39.6% *vs.* 50 events in 41.7%	NR
Abbassy *et al.* [6] (2017)	Insulin glargine plus glimepiride (Baseline *vs.* week 24)	10.35 ± 1.6 *vs.* 7.85 ± 1.29 (P<0.001)	219.6 ± 73.6 *vs.* 115.2 ± 62.4 (P<0.001)	312.71 ± 95.04 *vs.* 155.88 ± 56.61 (P<0.001)	37 events in 11.07%	No significant change (P>0.05)
Echtay *et al.* [26] (2017)	Insulin detemir+ OADs (Baseline *vs.* week 24)	9.7 ± 1.6 *vs.* 7.2 ± 1.0 (P<0.0001)	213.7 ± 60.1 *vs.* 120.3 ± 25.7 (P<0.001)	271 ± 65.3 *vs.* 158.1 ± 36.4 (P<0.0001)	2.0069 *vs.* 1.8556 per person year	80.4 ± 13.2 *vs.* 79.9 ± 12.5 (P<0.0001)
Tsukube *et al.* [30] (2016)	Glargine + SU	-0.96 (P<0.001)	NR	NR	3.3	NR
	Glargine + DPP-4i	-2.46 (P<0.001)	NR	NR	1.9	NR
	Glargine + BG	-2.76 (P<0.001)	NR	NR	5.2	NR
	Glargine + SU+ DPP-4i	-1.40 (P<0.001)	NR	NR	6.3	NR
	Glargine + BG+ DPP-4i	-1.34 (P<0.001)	NR	NR	4.1	NR
	Glargine + BG+ SU	-1.31 (P<0.001)	NR	NR	3.6	NR
	Glargine + BG + SU + DPP-4i	-1.34 (P<0.001)	NR	NR	3.9	NR
Odawara *et al.* [58] 2015	Glargine + OADs (Baseline *vs.* week 24)	-1.47	198.4 ± 62.3 *vs.* 136.1 ± 42.4	264.1 ± 85.6 *vs.* 196.7 ± 71.9	1.0	0.8
Kim *et al.* [59] (2015)	Glargine +/- OADs or Prandial insulin (Baseline *vs.* week 24)	9.2 ± 1.4 *vs.* 7.4 ± 1.1	NR	NR	17.6 (overall)	65.2 ± 10.3 *vs.* 65.5 ± 10.1

**Abbreviations:** BMI, body mass index; BG, biguanides; DPP-4i, Dipeptidyl peptidase-4 inhibitors; NR, not reported; OADs, oral antidiabetic drugs, SU, sulfonylureas.

**Table 3:** Key recommendations.

**S. No.**	**RECOMMENDATIONS**
1.	Early introduction of basal insulin therapy helps to achieve better control of glycemic variability, maintains β-cell function and slows down the progression of diabetes-associated complications.
2.	Glimepiride plus metformin combination show synergistic action in terms of glycemic control and better safety profile by reducing hypoglycemia, weight gain and cardiovascular profile compared to older generation SUs.
3.	Combining basal insulins to oral therapy (glimepiride plus metformin) appears to be effective in improving glycemic control and lowers insulin dose requirement.
5.	Different basal insulins have diverse properties, and their choice can depend on individual patient’s clinical condition and treating physician’s discretion.
4.	Second-generation basal insulin (BI) analogues (gla-300, degludec) have comparable glycemic efficacy with less hypoglycemia compared to first-generation BI analogues.
6.	Precautions to be taken when administering glimepiride and metformin combination to avoid hypoglycemia by employing appropriate doses of insulin.

## LIST OF ABBREVIATIONS

OADs: Oral Anti-Diabetic Drugs
T2DM: Type 2 Diabetes Mellitus
SUs: Sulfonylureas
DPP4i: Dipeptidyl Peptidase-4 Inhibitors
SGLT2i: Sodium Glucose Cotransporter 2 Inhibitor
FPG: Fasting Blood Glucose
NPH: Neutral Protamine Hagedorn
BOT: Basal Combined Oral Therapy
FDA: Food and Drug Administration
BI: Basal Insulin
